# Microplastics, a Global Issue: Human Exposure through Environmental and Dietary Sources

**DOI:** 10.3390/foods12183396

**Published:** 2023-09-11

**Authors:** Lucrezia Borriello, Marcello Scivicco, Nunzio Antonio Cacciola, Francesco Esposito, Lorella Severino, Teresa Cirillo

**Affiliations:** 1 Department of Veterinary Medicine and Animal Production, Division of Toxicology, University of Naples Federico II, Via Delpino 1, 80137 Naples, Italy; lucrezia.borriello@unina.it (L.B.); marcello.scivicco@unina.it (M.S.); nunzioantonio.cacciola@unina.it (N.A.C.); lseverin@unina.it (L.S.); 2 Department of Public Health, University of Naples Federico II, Via Sergio Pansini, 5, 80131 Naples, Italy; 3 Department of Agricultural Sciences, University of Naples Federico II, Via Università, 100, 80055 Portici, Italy; tcirillo@unina.it

**Keywords:** microplastics, human health, source, foods, food chain, trophic transfer, human exposure, fate, contaminants, toxicity

## Abstract

Plastic production has grown dramatically over the years. Microplastics (MPs) are formed from the fragmentation of larger plastic debris by combining chemical, physical, and biological processes and can degrade further to form nanoplastics (NPs). Because of their size, MPs and NPs are bioavailable to many organisms and can reach humans through transport along the food chain. In addition to the risk from ingesting MPs themselves, there are risks associated with the substances they carry, such as pesticides, pathogenic microorganisms, and heavy metals, and with the additives added to plastics to improve their characteristics. In addition, bioaccumulation and biomagnification can cause a cumulative exposure effect for organisms at the top of the food chain and humans. Despite the growing scientific interest in this emerging contaminant, the potential adverse effects remain unclear. The aim of this review is to summarize the characteristics (size, shape, color, and properties) of MPs in the environment, the primary sources, and the transport pathways in various environmental compartments, and to shed more light on the ecological impact of MPs and the potential health effects on organisms and humans by identifying human exposure pathways.

## 1. Introduction

Plastic has found wide use, replacing wood, paper, glass, and metal because of its characteristics: It is easy to work with, cheap, lightweight, and impermeable to water; has low thermal conductivity; and is an electrical insulator [1,2]. Its production has grown rapidly, from about 1.7 tons per year in the early 1950s to more than 350 million tons annually in the 21st century [3]. Hundreds of synthetic polymers are produced, including polyethylene (PE, ~28.5%), polypropylene (PP, ~16.7%), polyvinyl chloride (PVC, ~9.1%), polyethylene terephthalate (PET, ~8.1%), polystyrene (PS, ~6.1%), polyurethanes (PU), and polyamides (PA) [4]. The primary use of plastics is in food packaging; consumer products for industries and consumers, including single-use products; and semi-finished industrial products, for which significant volumes of PE, PP, PET, and PS are used. Construction is the second largest area of use, with PVC finding extensive use in plumbing fixtures. However, the textile industry also uses large volumes of plastics, with PA (e.g., nylon), PU (e.g., spandex), PE, and PP (insulating polyolefins) being used. Recently, the use of PET for nonwoven fabrics has been added [5]. Plastic products are used for very short periods and quickly become waste: packaging in less than 1 year, consumer products within 5 years of production, and products in the textile industry within 10 years. One exception is construction plastics, which are used for decades before becoming waste [6].

The amount of plastic to be disposed of yearly is a severe environmental problem [7], and its sustainable management has been included in the 12th Sustainable Development Goal (SDG). Disposal can be carried out in three ways: recycling, incineration, or release to the environment through dispersal or landfill storage. Recycling allows new plastic products to be produced from waste but requires adequate infrastructure and waste consisting solely of plastics [8]. Incineration is a disposal method that produces energy [9] and does not require special sorting procedures.

A series of additives are added to plastic products that modify their chemical and physical properties to improve specific characteristics such as color, flexibility, transparency, resistance, and persistence. These substances are not chemically bound to the plastic polymer, so they can be quickly released into the environment; only some flame retardants are polymerized with plastic molecules, becoming part of the polymer chain [10]. Additives are classified according to their function: inert fillers (silica), reinforcers (fiberglass), flame retardants (organobromine), dyes (copper phthalocyanines), plasticizers (phthalic acid esters), antioxidants (bisphenols), bioacids, and stabilizers. They complicate the disposal and recycling of plastic.

Approximately 60% of plastic waste is released into the environment, with almost 30 million tons per year [11], which is poorly managed. Despite being large producers, in high-income nations such as European countries, the United States, Australia, South Korea, Japan, and New Zealand, less than 10% of waste is mishandled. In contrast, 80% of plastic waste is released into the environment in developing countries such as in Southeast Asia. It is estimated that 4 to 12 million tons of plastic end up in the sea yearly [12], and its volume in the oceans is about 269 million tons [13]. Currents transport such waste and form huge plastic islands, the largest of which measures more than 1,500,000 km^2^ (three times the size of Spain) and is located in the Pacific Ocean [14], which is why the United Nations has included the protection of the sea and waterways against plastic pollution in the 14th Sustainable Development Objective [15].

Among the most commonly found debris in oceans and on beaches are bottle caps. Over the past three decades, beach cleanups have recovered more than 20 million plastic, aluminum, and iron bottle caps. This widespread presence is attributed to inadequate disposal methods and insufficient waste management technologies. Recycling rates for bottle caps vary from country to country, with only 20% in the United States, 40% in Europe, and a remarkable 90% in Japan. Lower recycling rates contribute to increased water pollution from bottle cap waste. In a 2022 study, Nikolaev investigated the physical properties of metal and plastic bottle caps to implement gravity separation, a process widely used in various industries due to its simplicity and environmental friendliness. This method leverages differences in particle properties such as density and size to influence their behavior in water. Specifically, if a particle’s density exceeds that of water, it sinks, whereas lighter particles float towards the water–air interface. The study examined bottle caps made of aluminum, iron, high-density polyethylene (HDPE), low-density polyethylene (LDPE), and polypropylene (PP), revealing notable differences in particle densities. Plastic particles had densities ranging from 0.752 to 0.956 g/cm^3^, whereas aluminum and iron particles exhibited significantly higher densities of 1.66–1.97 g/cm^3^ and 4.43 g/cm^3^, respectively. Consequently, in aquatic environments, plastic particles float, whereas metal particles sink. Experimental tests yielded terminal sedimentation velocities for aluminum (12 cm/s) and iron (18 cm/s) particles that were significantly higher than the velocities observed for plastic particles (ranging from 2.1 to 6.1 cm/s). Based on these findings, a simplified process flowsheet was proposed for the recovery of plastic and metal bottle caps from sandy shores, addressing the critical issue of coastal cleaning while enabling the retrieval of valuable materials [16]. In light of increasing environmental awareness and the importance of circular economy principles, the sustainability of packaging materials is paramount. Although the use of recycled polymer materials is on the rise, they often exhibit lower surface quality due to material heterogeneity, thermal degradation, and additives. In contrast, bottle caps are precision-engineered products with stringent quality requirements.

To assess the environmental impact and enhance the production process, a Life Cycle Assessment (LCA) was conducted in 2023 by Agarski et al. Specifically, this study aimed to improve the design of multi-component plastic caps for 19 L water bottles. The results demonstrated that the redesigned caps, with fewer components and reduced mass, had significantly lower environmental impacts across various parameters, including global warming, freshwater eutrophication, ozone formation, and terrestrial acidification. Additionally, modifications to the production machinery and tools not only reduced environmental impacts but also led to reductions in raw material usage and energy consumption, thus highlighting the economic benefits associated with environmental improvements [17].

Plastics have very long degradation times [18], and few organisms can feed on plastic polymers [19], except for the bacterium *Ideonella sakaiensis*, which can degrade PET [20], and some algae that can use plastic waste as a substrate. The decomposition of the plastic in the environment occurs mainly by photolysis, which causes oxidation and breakage of the polymer chains and makes possible the subsequent attack by bacteria. Long photodegradation times promote the hardening of and reduction in the flexibility of the plastic product [21], which facilitates the fragmentation of plastic waste due to mechanical stress and results in the production of MPs. Therefore, MPs derive from the fragmentation of larger plastic debris [14] through a combination of chemical, physical, and biological processes affecting the structural integrity of the residue [22]. Ultraviolet radiation causes the oxidation of the polymer matrix, resulting in the breakdown of the bond [23], which can also lead to the leaching of additives [24]. Plastic debris on beaches has a high availability of oxygen and direct exposure to sunlight, leading to rapid degradation [23]. MPs can further degrade to form nanoplastics (NPs) smaller than 0.1 μm [23]. Biodegradable plastics are considered a valuable substitute for conventional plastics. Still, they can also be a source of MPs [25], as they are composed of synthetic polymers and starch, vegetable oils, or specialized chemicals to reduce degradation times [14]. This degradation is only partial because the starch components of the bioplastic decompose but not synthetic polymers [23,25]. The aim of this narrative review is to provide a comprehensive and qualitative summary of existing literature on the environmental and food occurrence of MPs.

### 1.1. Microplastics Classification

There are controversies about the definition of MPs from the point of view of size [26], but several United Nations agencies categorize plastic wastes dispersed in the environment into megaplastics (larger than 1 m), macroplastics (1 m–25 mm), mesoplastics (25 mm–5 mm), MPs (5 mm–1 μm), and NPs (smaller than 1 μm) [27,28]. MPs are also classified according to the shape of their fibers, pellets, and fragments. Fibers have a much greater length of width and thickness; pellets are spheroidal, with three sizes of the same order of magnitude; and fragments are “flat” and irregular particles, with a much smaller thickness of length and width, which are of the same order of magnitude [29]. Finally, the color of MPs can depend on both the original color of the plastic object from which it comes and the process of degradation of the polymer, but it can also depend on external contaminants. Black, blue, white, transparent, red, and green are the most common [30]. Color plays an important role, especially in aquatic studies, as some species ingest MPs based on color preference behavior; in particular, marine animals more frequently consume transparent or white MPs. In addition, it may indicate the number of contaminants absorbed by MPs; for example, yellow or black MPs are those contaminated by persistent organic pollutants [31]. MPs are complex particles with specific characteristics that are subject to change over time and that affect their degradation time, movement, and interactions with the environment [32].

A further classification of MPs is based on the origin in two categories: primary and secondary. The former is made of tiny particles intentionally produced in “micro” size at an industrial level and dispersed in the environment accidentally or through their use. This category includes microspheres in personal care products, such as facial detergents, cosmetics, soaps, and different kinds of toothpaste [33]; medicines such as drug carriers; and plastic pellets lost during the transport or processing of plastics [23]. MP “scrubbers” have replaced pumice, ground almonds, and oatmeal, which were previously used for exfoliating cleansers [34], and, since their introduction to the market, use of them has increased exponentially [34]. The proportion of primary MPs in the environment is lower than that of secondary MPs, except in some large lakes in the United States, which are located close to large industries [35].

Secondary MPs, conversely, are produced by the degradation of plastics exposed to weathering, even though this category also includes fibers released during the washing of synthetic fabrics [22] and the wear and tear of vehicle tires [36]. Fibers are the most abundant form [22] due to the continuous abrasion of clothing and upholstery made from synthetic fabrics and release from washing machine effluents [3]. It has been estimated that more than 1900 fibers can be released per item during washing; in this context, textile factories could be a significant source of environmental release.

### 1.2. Transport of Microplastics

MPs are light and can be easily transported from waterways [37]; in fact, it is estimated that between 70 and 80% of marine plastic is transported by rivers, with the entrance of MPs occurring through urban and industrial discharges. However, the latter are subject to standard wastewater treatment [20]. In fact, most MPs do not precipitate by density on the bottom of the system and are released into the environment. MPs are much denser than air and tend to precipitate on land or at sea [38]; however, their meager weight allows wind to transport them as atmospheric dust, even over long distances [39], going on to contaminate even remote areas with very little or no anthropogenic activity [40] and making them a ubiquitous contaminant. Atmospheric deposition can be a potential source of MPs for freshwater systems and the marine environment. The first scientific document on the presence of MPs in atmospheric particulates was published in 2015 [41]. Their origin stems from tire wear [42] and plastic waste due to mechanical stress [43]. Several studies have estimated the production of 20–30 mg of micro- and nanoparticles per km covered by an average tire [44] and even more for heavier vehicles. In addition to the fragmentation that occurs in the atmosphere, different materials splinter during use, leading to the formation of microscopic particles in the atmosphere, as in the case of the fibers released from clothes during washing [45].

About 80% of MPs at sea come from products consumed on land; only 20% come from activities at sea: from the wear of fishing nets and plastic parts of ships, spills while transporting pellets and plastic products, and illegal disposal of plastic waste at sea. Their presence has been documented in marine ecosystems around the world [46]; this presence in environments far removed from human activity is due to sea currents that carry them far from the point of release [33], and storm waves bring them back to the coasts [47]. The higher density compared to water allows MPs to settle on the seabed, in particular, particles of PVC, PET, PU, and PA or materials with additives such as silica, which precipitates even lighter polymers such as PE, PP, and PS. Furthermore, the colonization of plastics by biofilm-forming microorganisms causes weight gain and facilitates deposition [48].

## 2. Materials and Methods

### 2.1. Literature Search Strategy

A comprehensive search for relevant literature was conducted using Google Scholar as the primary search engine. The following keywords were used in various combinations to identify articles related to microplastics, human health, sources, food contamination, trophic transfer, human exposure, fate, contaminants, and toxicity:

- “Microplastics”
- “Human health”
- “Source”
- “Foods”
- “Food chain”
- “Trophic transfer”
- “Human exposure”
- “Fate”
- “Contaminants”
- “Toxicity”

### 2.2. Inclusion and Exclusion Criteria

The search was not limited by publication date and only articles in English were included. Articles were included in this narrative review if they met the following criteria:

1. Articles that focused on the relationship between microplastics and human health;
2. Studies that examined the sources and pathways of microplastic contamination in the environment, primarily related to the food chain;
3. Studies assessing microplastics’ toxicity and potential health impacts on human populations.

### 2.3. Study Selection and Data Extraction

The initial search yielded a total of 5490 articles. After removing duplicates and applying the inclusion and exclusion criteria, 183 articles were selected for detailed review, of which 163 were original research and 20 were reviews.

## 3. Sources of Exposure to Microplastics

MPs are ubiquitous in the environment; they have been detected in air, soil, freshwater, drinking water, oceans, aquatic and terrestrial biota, food, placenta, and human feces [49]. Humans are constantly exposed to these substances, but some forms of exposure are much more significant than others. The primary source is gastrointestinal through the ingestion of food and drink, the secondary source is respiratory through the MPs present in the air, and the third source is absorption through the dermis, although it has never been observed, unlike with other organic contaminants, and would still be marginal compared to other forms of exposure. Their presence in so many environmental compartments inevitably implies daily exposure, which has increased steadily and annually with increased production and use of plastic; this has attracted the attention of the scientific community, the public, and political authorities such as the European Commission, the European Chemicals Agency (ECHA), and the World Health Organization (WHO), which recognize them as a potential hazard to human health and wildlife [50]. The assessment of these risks is challenging. Some studies have been conducted to assess the impact of MPs, taking into account stability under various environmental conditions [51], adsorption or leaching of chemicals [52], and cellular biological effects [53], but no risk assessment framework considers the variety of sizes, shapes, and chemical properties.

Furthermore, the different exposure assessment methods used do not allow for comparison between studies [54]; therefore, although human exposure to MPs is unavoidable, the potential risk of such exposure has not yet been thoroughly and definitively investigated. Identifying even the smallest particles is necessary to assess exposure, as several studies have shown that the toxicity is related to the particle size [55]. However, the most widely used methods for identifying MPs use FT-IR or Raman spectroscopy, which can only confirm MPs larger than 10 μm [56]. At the same time, the most marked toxic effects were observed mainly for much smaller particles.

### 3.1. Microplastics in Soil

Agriculture is undoubtedly one of the main anthropogenic activities responsible for MP pollution in the soil, because of both sewage sludge used to modify the soil and agricultural plastics, such as plastic mulches, used to increase crop yields [57]. Each year, 125 to 850 tons of sewage sludge are added to European agricultural land [57]. As mentioned above, light macroplastics and MPs can be carried by the wind, whereas heavier macroplastics end up in deep layers and remain in the soil [58]. Fragmentation of plastics is hampered by reduced exposure to air and UV rays, which makes soil a reservoir for MPs [59]. Their potential impact on soil has only gained attention since 2016, although MPs have the potential to alter soil geochemistry and interact with soil biota [60]. The studies were mainly conducted on the ingestion of MPs by soil biota. In particular, Lwanga et al. (2016) studied the survival of the earthworm *Lumbricus terrestris* after exposure to different concentrations of MPs. Laboratory experiments have been carried out, adding MPs and organisms to soil samples, but field experiments are scarce [61]. In 2012, Rillig identified the problem of MP pollution in soil [62,63], but the number of soil studies is still small compared to aquatic habitat studies [64]. PE and PP are the most frequent polymers in soil, but smaller amounts of PVC and PET are also found. Such polymers could be environmentally friendly if it were not for additives that increase their toxicity, such as phthalic acid esters (PAEs) [32], which are widely used in films used in agriculture and have also been found in fruits and vegetables [65]. In soil, macroplastics fragment into MPs and NPs, which can absorb heavy metals or release organic pollutants into the soil, such as PAEs, which pose a risk to soil biology and human health [66]. Interestingly, MPs and PAEs have also been found on agricultural land where fertilizers containing plastic have never been used [67].

Macroplastics and MPs enter soils through mulching films, municipal waste, biosolids (sewage sludge), plastic-coated fertilizers, and atmospheric deposition [68]. Among these, mulch films are the most significant source [69]. Plastic mulching improves water and nutrient resource efficiency, provides thermal insulation, anticipates planting and harvesting [70], and uses pesticides more efficiently [71]. For these reasons, industries have promoted their use [66], neglecting the resulting pollution. These films are very thin (about 8–50 mm thick) and very difficult to extract at the end of the season [72], so they have accumulated in agricultural soils and, over time, fragmented into macro-, micro-, and nanoplastics [66].

Biosolids can replenish organic matter in farming systems [73], even though it is well known that they can contain metals and organic pollutants [74]. It is evident that they also have significant amounts of plastics [75], as between 70 and 99% of the MPs in domestic wastewater are recovered in sludge during water treatment [76], with MP concentrations of about 103–105 particles/kg. After repeated sewage sludge applications to agricultural land, many MPs accumulate in the soil [57,64]. Municipal solid waste dumps contribute to this contamination, affecting the ground and involving the underlying groundwater [64]. China is among the world’s largest producers and consumers of plastics, as about 2.6 million tons of agricultural film are used annually. The terrestrial environment has a much higher contamination of MPs than marine environments [57,58], and this contamination varies at different soil depths [68]. Liu et al. (2018) showed that the amount of MPs in more superficial areas of soil is higher than in deep soils [68]. Macroplastics affect soil structure, limiting rainwater and irrigation water infiltration, soil water retention capacity [77], and soil aeration [77], affecting root growth and plant productivity [63]. In addition, agricultural plants likely absorb MPs, which then enter the food chain [78].

### 3.2. Microplastics in the Aquatic Environment

MPs present in the sea have various origins [38]. Fishing is a direct source of MPs in the marine environment, but it is not the most significant [79]; most of them come from terrestrial sources, such as the degradation of stranded waste, freshwater streams, wastewater discharges, and atmospheric deposition of MPs into the air [14]. Identifying MP polymers was crucial in determining potential sources [80]. The predominant form is fiber, which is found in marine sediment [81], surface waters, and the water column [82], as well as in biota [83], which suggests that the source is wastewater as a result of laundry activities. For quantity, there are fragments mainly generated by the fragmentation of large plastic debris [30]. The different colors of the MPs found in aquatic environments suggest additional sources. For example, transparent fibers could result from broken fishing lines or nets.

In contrast, colored fibers are more likely to result from the fragmentation of plastic products, such as clothing and packaging [84]. The polymers with which the plastic is made involve differences in composition, density, and shape, so MPs are found at different points of the water column: The low-density ones will be on the surface [85], unless their surface is colonized by algae invertebrates, which increase the density of the particles and encourage the sinking of floating MPs [23]. The formation of biofouling increases the density of plastic [86]. High-density MPs, such as PVC, PE, and PA, settle on the bottom, but their determination is hampered by cost and sampling difficulties [87].

## 4. Microplastics in Processed Foods

MPs are found in foods such as seafood, vegetables, fruits, and rice [88], but also in processed foods such as bottled water, milk, beer, sugar, honey, table salt, and beverages [89]. Their presence in food may come from packaging [90], as confirmed by studies of some food products [91]. Thus, it is clear that processed foods are more likely to contain MPs than unprocessed foods, and for that reason, these foods contribute more MPs to human consumption [92,93]. Many in vitro studies have revealed interactions of MPs with gut cells and with food components such as proteins and lipids in the digestive tract [94]; however, human consumption of MPs through the diet has been evaluated by very few studies. Cox et al. (2019) estimated the number of MPs that humans consume through food and inhalation, but the potential health risk has not been assessed [95]. Another study calculated the probabilistic exposure to MPs over a human lifetime by considering the number of MPs in the intestinal tract, body tissues, and feces, but the chemical risk associated with MPs consisting of various types of polymers was not considered.

In these foods, the most common polymers are PE (22.8%), PET (22.0%), and PP (19.0%), which are used precisely for food packaging and plastic bottles [9]. Sobhani et al. (2020) [96] highlighted that MPs could be released during the opening of packaging or the opening and closing of plastic water bottles [97]. In addition, raw materials of processed foods may be subject to contamination by MPs present in the environment. As mentioned earlier, the chemical characteristics of MPs can be examined to identify potential sources; Zhang et al. (2021), in their study, analyzed MPs in human excrement and found that PP and PET accounted for nearly 80% of the MPs detected [2]. The chemical similarity of these MPs to those found in processed foods suggests that the risk of exposure to MPs from consuming processed foods cannot be overlooked.

### 4.1. Microplastics in Table Salt

Salt is undoubtedly an insidious form of MP intake, as it is a widely used ingredient in the diet of the world population and is present in almost all packaged foods. The daily salt consumption of 5 g/day recommended by the International Health Organization (WHO, 2007) is largely exceeded, so it can become a significant source of exposure. Salt is naturally present in food, but 75–80% is added [98]. Some foods are particularly rich in salt, such as processed meat products, with 0.5 to 4 g per 100 g product [99]; dairy products, with 0.6 to 2.5 g per 100 g product [100]; and some cereal, potato, or peanut “snacks,” with a content of 0.5 to 7 g per 100 g of product [101]. Soy sauce is widely consumed in the Far East and has a content of 12.5–20 g per 100 g of product [102]. In 2020, world salt production reached 270 million tons; 4% is destined for food. It is extracted from seawater (40%) or underground deposits (60%). The discovery of MPs in salt dates back to 2015 [103], and interest in the issue has increased since then. In mine salt, the presence of MPs is limited and linked to the pollution of mining techniques.

On the other hand, sea salt has a much higher content, as it depends on the MP content of seawater [104]. Polymers found in seawater mainly include PE and PP, which are frequently found in sea salts. Food salt may undergo industrial refining to reduce the impurity content [105], but whether this process can eliminate or at least reduce the MP content has not been studied.

### 4.2. Microplastics in Sugar

Among the most consumed foods in the world is sugar, which will increase further in the coming years due to the increasing demand for drinks, biscuits, cakes, and jams [106], but it too can contribute to human exposure to MPs. One study analyzed the presence of suspicious MP particles, including fibers and fragments in refined, unrefined, and powdered sugar samples. However, the polymer composition of the identified particles has not been confirmed, and there is no clear description of the origin of the samples analyzed or the analytical procedures adopted [107].

Afrin et al. (2022) examined the presence of MPs in sugar samples obtained in Dhaka, Bangladesh [108], where the sugar industry plays a key role [109]. All the particles found that were suspected of being MPs were quantified, classified, and analyzed; a risk assessment was carried out to investigate the contribution of sugar to MP contamination in Dhaka. Five commercial and two non-branded sugar brands were collected and examined. MPs had contaminated all samples, and their polymer composition was confirmed by FT-IR. All the MPs identified in the sugar samples were high-density PE. The number of plastic particles/kg of sugar was, on average, 343.7 ± 32.08 (mean ± SEM), with the lowest and highest concentrations recorded being 183.0 and 669.0, respectively, with a coefficient of variation of 42.77%. As for the size of the identified particles, there was an apparent heterogeneity; however, a higher frequency of MPs below 300 μm was observed, classified as “small MPs” (64%). Microfibers and spheres were the most frequent types of plastic (38.4% and 7.9%, respectively). In terms of color, black, pink, blue, and brown predominated over the other colors (white, yellow, orange, red, and transparent). From the evaluation carried out, it was estimated that the consumption of sugar in the city of Dhaka may involve the ingestion of millions of tons of MPs per year (from 2.4 to 25.6 tons, with an average of 10.2 tons) of different sizes, shapes, colors, and polymer compositions. Contamination occurs during the various stages of production, such as processing and purification of sugar cane, refining, and packaging. During these phases, dispersed MPs in industrial environments can enter or be released from the machinery used. However, in some stages, contamination is even more likely. For example, in the drying phase, large dryers are used to reduce the moisture content of sugar; these, if poorly managed, can provide a stream of contaminated air. Although filters are recommended for these dryers, the absence or uneven maintenance of them contributes to poor flow efficiency, and filter cleaning alone is usually insufficient. In addition, the passage of sugar occurs through plastic ducts that can release particles. Finally, in the storage phase of the final product, the sugar is placed in bags that can release fibers and plastic fragments and end up in the sugar. Recently, nine types of polymers have been classified as potentially hazardous to health by Yuan et al. (2022) [110], although the effects on humans have not yet been clarified. As evidenced by Hirt and Body-Malapel (2020), studies on animal models suggest that MPs induce disturbances in intestinal homeostasis, alterations in intestinal permeability, deregulation of the gut microbiota, and changes in the levels of immune cell recruitment or cytokine secretion [111].

### 4.3. Microplastics in Bottled Water

MPs have recently been detected in bottled water [112,113] packed in plastic and glass bottles. Given the large consumption of mineral water, this topic has attracted public interest and media attention. Schymanski et al. (2018) examined the contents of MPs in 38 water samples in PET bottles, glass bottles, and beverage cartons, of which there were 26 PET bottles (15 multipurpose bottles and 11 disposable bottles), 9 glass bottles, and 3 beverage cartons; the samples were taken from the German market [111]. A micro-Raman spectroscope was used to identify MPs after water filtration. The study found 118 ± 88 particles per liter in the multipurpose PET bottles, 84% of which were PET particles, thus suggesting that the packaging material is a source of MPs. In disposable PET bottles, only 14 ± 14 particles per liter were detected; again, most of the particles were PET. Only 11 particles per liter were found in the three coated cartons.

In comparison, 4 to 156 particles per liter were found in the glass bottles. Since most of the particles in the PET bottled water were identified as PET, the authors concluded that the MPs come from the bottles themselves. In addition, the authors hypothesized that the material of multipurpose bottles was more stressed than that of disposable bottles, leading to a more significant release of MPs into the water.

In another study, Oßmann et al. (2018) investigated 32 bottled water samples (12 in multipurpose PET bottles, 10 in disposable PET bottles and 10 in glass bottles, of which 1 was disposable and 9 were multipurpose glass) by filtration and use of micro-Raman spectrometry [110]. Unlike Schymanski’s measurements, which were limited to particle sizes greater than or equal to 5 μm, Oßmann determined particle sizes of up to 1 μm. The number of MPs in the water ranged from 2649 ± 2857 per liter in the disposable PET bottles, to 4889 ± 5432 in the multipurpose PET bottles, to up to 6292 ± 10,521 per liter in the glass bottles. The predominant polymer type in the PET bottles was PET. In contrast, polymers such as PE and styrene-butadiene copolymer (SBC) were identified in the glass bottles. This study confirmed that bottled water contains MPs and other beverages. Contamination arises from packaging because PET and PE polymers, with which bottles are made, are the most frequently found polymer types in bottled water.

## 5. Microplastics in Seafood

MPs are present in almost 700 aquatic species worldwide, including sea turtles, penguins and other crustaceans, but also in bivalve mollusks [114] such as mussels, *Mytilus edulis* [115] and clams, *Ruditapes philippinarum* [116] and in many widely consumed fish species such as European hake, Merluccius [117], sardine, *Sardina pilchardus* [118] mackerel, *Scomber scombrus* [119], common sole, Solea [120], bluefin tuna [121], flounder, *Pleuronectes platessa* [122] and European anchovy, *Engraulis asicolus* [118]. Numerous factors favor the ingestion or assimilation of MPs by marine organisms, such as size (smaller ones are more bioavailable), density (a higher amount of MPs leads to a higher probability of ingestion and/or adsorption) and color (some colors attract more groups of organisms than others) [123]. On the other hand, MPs release volatile organic compounds, such as dimethyl sulfide (DMS), during their aging process in the marine environment; this compound is also found in algae and represents an olfactory mark, so some organisms ingest MPs, mistaking them for prey [124]. 1 to 5 MPs per specimen have been found in fish, lower than bivalve shellfish, which are filter organisms and tend to accumulate MPs [125].

### 5.1. Microplastics in Mussels

Mussels are filtering organisms that treat large volumes of water (on average 7–8 L of seawater per hour); as a result, they accumulate and concentrate many pollutants in the sea. They are ideal for monitoring the state of environmental pollution because they have a wide geographical distribution, are sessile, so sampling is relatively easy, tolerate changes in salinity, resist stress and accumulate different types of pollutants [126]. Mussels can contribute to transferring MPs to organisms at higher trophic levels [127]. For these reasons, studies have been conducted worldwide to monitor the pollution of MPs with mussels. In particular, Phuong et al. (2018) found eight different plastics in the blue mussels (Mytilus edulis) of the French Atlantic coast, with a predominance of PE and PP [128]. Most of the MPs were fragments and an average of one particle per mussel was detected. In Norwegian waters, mussels from the Barents Sea were compared with mussels from Oslofjord [129]. The mussels contained 13 different polymers; an average of 1.5 particles/individual was detected, with fibers representing 83% of the identified particles. Once ingested by mussels, MPs can be transferred from the intestine to the circulatory system and remain in the body for 48 days [130]. Since mussels accumulate MPs, are found at the base of the food network and are widely consumed by organisms at higher trophic levels, including humans [131], they can transport MPs along the food chain [132]. MPs accumulate mainly in the gastrointestinal tract, which in organisms such as mussels is not removed before consumption, unlike fish [133]. Consequently, MPs in fishery products may affect food safety and human health [126]. Seafood should ideally be reared or harvested in unpolluted areas; however, as it is increasingly difficult to find pristine marine habitats, these organisms can become high-risk foods.

### 5.2. Microplastics in Fish

MPs have been found in different foods [134], but fish is an important source; in fact, depending on consumption habits, a person can ingest 1 to 30 MPs per day by eating fish. Ingestion by fish may occur directly or indirectly by consuming other organisms containing MPs [135]. After ingestion, MPs accumulate in the digestive systems, including the stomach and intestines [136], but may also translocate into other tissues, such as gills, liver and muscles [137], where they can cause multiple adverse effects on the health of the organism, such as infertility, growth retardation, internal or external injuries and blocking of body traits [138].

## 6. Transfer to the Food Chain

The entry of MPs into the food chain takes place through marine organisms, in particular, shellfish [139], but also through drinking water [140], table salt [141], fruits and vegetables [142], and other foods [143]. Studies have focused more on marine organisms that, as mentioned above, can ingest MPs directly or indirectly through prey and breathing. Regardless of the route, the intake of MPs may have adverse effects on marine organisms [144] due to the physical retention of MPs in the digestive tract and the chemical leaching of plastic additives in tissues [145]. The concepts of bioaccumulation and biomagnification are related to chemical contamination [146], but the terminology has also been adopted for MPs [147]. The absorption of a contaminant, such as MPs, from the environment by contact, ingestion, or respiration is called bioaccumulation, and such absorption must be greater than the ability of an organism to eliminate it [148]. Bioaccumulation and trophic transfer of a contaminant result in biomagnification [149], which is the increase in the concentration of a contaminant in an organism relative to the concentration present in its prey. Trophic transfer of MPs has been confirmed under laboratory conditions from mussels (*Perna perna*) to crabs (*Callinectes ornatus*) and pufferfish (*Spheoeroides greeleyi*) [150] and from algae (*Fucus vesiculosus*) to periwinkles (*Littorina littorea*) [151]. The adverse effects of MPs on marine organisms have been studied via laboratory and field experiments. A study conducted on the blue mussel Mytilus edulis revealed that smaller MPs tend to accumulate in the tissues of more than large particles and can translocate from the intestines to the circulatory system within three days and persist for more than 48 days [130]. In addition, exposure experiments have been conducted to assess the impact of MPs alone or in combination with toxic chemicals, including heavy metals, PCBs, PAHs, and polybrominated diphenyl ethers (PBDEs) [152]. The mechanisms of MP ingestion, translocation, and bioaccumulation in shellfish, including mussels [153], oysters [138], clams [154], and crabs [150], have been investigated. Li et al. (2016) studied MPs in mussels (Mytilus edulis) from the coast of China, and fibers were the most common MPs, followed by fragments [153]. Many other studies have confirmed that the most frequently ingested particles are fibers, which are the most abundant in the aquatic environment (66–71% of the total count), followed by fragments and pellets [83]. Studies on MP ingestion have mainly focused on invertebrates. Lusher et al. (2013) found MPs in 36.5% of fish of 10 species from the English Channel. The main polymers were polyamides and polyesters used extensively in fisheries [155]. These results overlap with those from the North Pacific Central Gyre reported by Boerger et al. (2010), where one-third of the fish caught contained small plastic fragments. Individuals of the most captured species, *Myctophum aurolanternatum* and Myctophidae, had an average of six plastic pieces with sizes of 1–2.79 mm [156]. Myctophidae is a mesopelagic family that preys on plankton. The color of most of the ingested plastic (white, clear, and blue) is similar to that of the plankton species inhabiting the central North Pacific, so it has been hypothesized that Myctophidae ingests MPs by mistaking them for their natural food source [156]. The toxicological effects of such ingestion have not yet been thoroughly investigated, but if they are unable to eliminate MPs, they will tend to accumulate. Myctophidae are preyed upon by tuna, squid, odontocete whales, seabirds, and fur seals [156], which will ingest MPs through their prey.

The whale shark (*Rhincodon typus*) is an endangered species that, like many other aquatic species, may be exposed to the ingestion of microplastics (MPs) and macroplastics due to its filtering activity at the sea surface. Increasing anthropogenic activity in areas inhabited by this species has resulted in significant pollution from both chemical contaminants and plastic debris. Whale sharks spend a significant portion of their day feeding near the water’s surface, where they may inadvertently ingest floating pollutants and plastic debris. These plastics can act as carriers of persistent organic pollutants (POPs) due to their high adsorption capacity for hydrophobic organic chemicals. In a 2017 study conducted by Fossi et al. in the Gulf of California, an ecotoxicological investigation of whale sharks was carried out using skin biopsies to assess their interaction with MPs and related contaminants. The study examined levels of organochlorine compounds (PCBs, DDT), polybrominated diphenyl ether plastic additives (PBDEs), and related biomarker responses (CYP1A). Skin biopsy samples collected in January 2014 revealed the following mean concentration values for target contaminants: 8.42 ng/g bw for PCBs, 1.31 ng/g bw for DDTs, 0.29 ng/g bw for PBDEs, and 0.19 ng/g bw for HCB. The average density of MPs in zooplankton/microplastic surface samples showed values ranging from 0.00 items/m^3^ to 0.14 items/m^3^, with the most abundant polymer being polyethylene (35%), which is also the most abundant polymer in plastic waste worldwide. The very short food chain of the whale shark may explain the lack of increased biomagnification of higher chlorinated contaminants, whereas the higher abundance of lower chlorinated PCBs is consistent with the fact that the surface waters of the oceans are rich in this contaminant. In addition, dicofol is an organochlorine pesticide chemically related to DDT that is used against the red spider mite. Finally, BDE-209 is used as a flame retardant in several plastic polymers and, in general, its distribution through the water column decreases with depth; the unusual levels found in the samples could have resulted from the surface-filtering activities of this species, so its presence in the tissues of whale sharks has been linked with the ingestion of plastic debris. In addition to direct ingestion, whale sharks may indirectly ingest MPs through the consumption of prey that could be contaminated with MPs. Addressing the issue of marine litter in the Gulf of California is therefore imperative to mitigate potential negative impacts on the unique biodiversity of this region [157].

## 7. Effects of Exposure to Microplastics

The discovery of MPs in the human body is very recent; they have been found in the colon, lungs [158], liver [159], kidney, spleen [159], blood [160], placenta [161], and breast milk [161], and this has increased the concern about this emerging contaminant. MPs entering the body cannot be excreted with urine; to avoid eliminating macromolecules such as proteins, filtration of renal glomeruli does not allow for the excretion of particles larger than 4.2 nm in diameter. However, organic additives such as bisphenols (antioxidants), phthalic acid esters (plasticizers), and organobromine (flame retardants) can be considered markers of MP exposure and can be found in urine. Some experiments on mice have shown that MP intake can release these substances into the body [162]. MPs pose a risk to biota, as their size and presence in pelagic and benthic ecosystems make them bioavailable to many marine organisms [25,34], especially those at low trophic levels that cannot distinguish between plastic particles and food. MPs constitute a mechanical hazard to tiny organisms [34]; plastic fragments can hinder the passage of food through the intestinal tract or cause pseudo-satiety with reduced food intake [163]. Plankton forms the basis of the marine food chain and has dimensions similar to those of MPs [52], so the latter are regularly traded for food and ingested [164]. Exposure of some marine animals to known concentrations of MPs has caused an increase in mortality [165], a reduction in food intake due to the “filling effect” due to mechanical action on digestive receptors [166], and a reduction in the growth rate of bivalves [167]. In addition, they cause damage to the gastro-enteric tract of *Danio rerio* [168]; in fact, MPs accumulate in the digestive tract [132], causing localized inflammatory phenomena and alterations in biological activity [145]. No animal has the enzymes necessary to degrade plastic polymers [23]; as a result, they remain blocked in the body and pass from one trophic level to another through predation [169]. One of the studies that evaluated human exposure to MPs estimated that 39,000 to 52,000 particles are ingested per year and that bottled fish and water are the primary food sources [95]. The recently studied [170] effects of MPs on humans include oxidative stress caused by the interaction of cells with the surface of particles, whose molecules are partially oxidized by degradation. This interaction causes an inflammatory response, producing reactive oxygen species such as peroxides and radicals, which fail to degrade the particle and damage the cell [171]. The possibility that MPs may induce an inflammatory reaction has led to the hypothesis that they could cause an increase in blood coagulability; increased activity of osteoclasts, which are the cells responsible for bone resorption; loss of bone mass; and interference with the immune system [136]. The health effects of MPs depend upon their location in the body as well as the properties of the polymers, particle sizes, and shapes. Factors such as MP concentration, exposure duration, surface modifications, and the presence of additives also play crucial roles. In mammalian studies, particularly in mice, these health risks have been assessed, revealing higher anxiety levels, reduced anti-predatory responses, and various other toxic effects. These toxic effects encompass oxidative stress and inflammation in the gastrointestinal tract, changes in liver metabolism (including abnormal amino acid metabolism), diminished reproductive capacity (including reduced fertility in males, decreased ovarian capacity, apoptosis of granulosa cells, and reduced sperm motility), and impaired behavior marked by depression and decreased food intake frequency [172]. However, it is unclear whether the dose used in these studies represents the actual human exposure to MPs. MP contamination in all ecosystems implies a high probability that biota and humans are exposed; however, the use of different methodologies (e.g., digestion and extraction from environmental matrices, visualization and use of spectrophotometric methods for identification) hinders the comparison of the various studies [173].

## 8. Toxicity

In addition to the potential adverse effects of ingesting MPs themselves, risks could arise from (a) additives released by MPs and (b) pollutants that adhere to them. The toxicity of a substance is linked to its ability to cause harmful effects; the most dangerous chemicals are those that can cause cancer and DNA mutations, have toxic reproductive effects, or accumulate in the food chain, such as endocrine disruptors. The most affected parts of the body are the liver, kidneys, heart, nervous system (including the brain), and reproductive system [174]. Plastic additives have a high and recognized toxic potential for humans and animals, with carcinogenic, neurotoxic, and endocrine-disrupting effects [10]. They can affect the endocrine system, impacting reproduction, development, and carcinogenesis [175]. Additives such as phthalates (PAEs) and bisphenol A (BPA) are endocrine disruptors and can mimic, compete with, or disrupt the synthesis of endogenous hormones [24]. In addition, chemicals that adhere to the surface of plastic fragments may be transferred to the tissues of organisms [176] and, through biomagnification, may accumulate in increasing quantities, increasing the risk of toxic effects for animals at the apex of the food pyramid and for humans [177]. BPA and PAEs are used to produce household products and food packaging and affect the functioning of organs that respond to hormonal signals; they can mimic natural hormones, antagonize their action, alter synthesis and metabolism, or change the expressions of specific receptors. There is evidence that these additives are linked to a wide variety of diseases, including cancer (breast, prostate, and testicle); genital malformations; infertility; metabolic disorders; and disorders related to asthma and autism [174]. In particular, BPA is both an estrogen agonist and an androgen antagonist and affects reproduction and development. It is a plasticizer used in the production of food packaging [178], as it contributes to prolonging the shelf life of food and beverages [178], but it has also been associated in several studies with obesity, cardiovascular disease, reproductive disorders, and breast cancer [174]. For this reason, BPA has gained increasing attention over the past decade, especially regarding human safety, as materials such as this one that come in contact with food could easily transfer from the packaging to the food [179]. PAEs are plasticizers added to give plastic polymers flexibility and elasticity [180]. They are a family of organic molecules composed of 10 diesters of specific phthalic acid with low water solubility [181]. Some of them are endocrine disruptors, and limits have been set in the European Union for toys and other children’s products (Directive 2005/84/EC) as well as for their use in food packaging (Commission Regulation (EU), 10/2011).

Finally, MPs can absorb a wide variety of persistent organic pollutants (POPs) from the environment, such as polycyclic aromatic hydrocarbons (PAHs), polychlorinated biphenyls (PCBs), and polybrominated diphenyl ethers (PBDEs), insecticides, pesticides, and industrial chemicals [182]. Hydrophobic particles such as plastic can easily absorb these non-polar molecules. MPs also absorb heavy metals, including mercury, cadmium, lead, aluminum, chromium, manganese, iron, cobalt, nickel, and zinc [183]. Plastic has been associated with 78% of the pollutants listed as a priority by the US EPA, so it can be considered a “cocktail of chemicals” that can become bioavailable to organisms [50]. Studying MPs in combination with other pollutants is crucial to understanding the behavior of plastic waste in the surrounding environment.

## 9. Insights and Observations

This review examined the main characteristics of MPs present in the environment. In particular, among those present in soil, the most abundant polymers are PE and PP, which can result from waste improperly released into the environment or MPs transported by air or watercourses but mainly through mulch films and sewage sludge used for agriculture. In the aquatic environment, the predominant form is fiber, and fishing activity contributes to only a small extent of the accumulation of MPs in the sea, as most of them derive from land through inadequate waste disposal and atmospheric deposition of MPs in the air and freshwater streams. Soil and sea contamination leads to contamination of plant products such as fruit and vegetables and fish products, but the presence of MPs has also been well documented in many processed foods, including salt, sugar, and bottled water. Contamination occurs during various stages of production, processing, and packaging, and, therefore, the predominant polymers are those in packaging. MPs are diffuse and persistent contaminants that accumulate in the environment and may compromise food safety or increase the spread of pathogens. MPs compromise soil geochemistry and interaction with biota and affect soil structure, limiting rainwater and irrigation water infiltration, soil water retention capacity, and soil aeration, affecting root growth and plant productivity. They pose a threat to organisms that ingest them by obstructing the passage of food through the intestinal tract or causing pseudo-satiety, resulting in reduced food intake. Moreover, they are detrimental to human health, carrying contaminants that act as endocrine disruptors, which is a matter of paramount concern. Pre-consumer efforts, namely, the reduction in and replacement of plastic products, and post-consumer approaches, namely, the disposal, collection, and recycling of plastic waste, are essential to reducing the number of macroplastics and MPs entering the environment. Awareness of the issue needs to be raised in order to establish certain policies and regulations to reduce plastic pollution. Effective policies should, therefore, motivate both industries and individuals to collaboratively reduce plastic pollution. Mitigating plastic pollution is a global challenge that can be tackled by reducing the amount of plastic produced and marketed, replacing plastic with alternative materials, implementing recycling projects, and reducing post-collection environmental losses.

## 10. Conclusions

We are in the “plastic age,” and although various measures have been taken to reduce plastic pollution, this environmental problem cannot be solved quickly. It is a priority to understand the toxicity mechanisms of MPs and the kind of damage they can cause. It is hard to tell which particle size causes the differences in toxicity since they seem to obey the rule of “the smaller the particle size, the greater the toxicity,” but the opposite is true in some tissues and organs. NPs can cross some barriers and contact tissues, whereas MPs are blocked and fail to exert their toxic effects.

Human health risk assessment requires further investigation; it is essential to unify sampling protocols, define analytical procedures, and standardize laboratory test conditions and the expression of results to allow for greater comparability between studies and their reproducibility. In addition, the production of standardized reference materials for all major types of polymers is necessary, as the diversity of industrially produced polymers is among the main limitations that do not allow for an accurate estimation of human exposure to MPs. Most studies use spheres of only one type of polymer, polystyrene (PS), so whether the effects observed with these particles can be extended to other polymers is not evident. In addition, most studies have concentrated on particles larger than 1 mm. At the same time, NPs (<1 μm) have shown significantly different physical and biological effects, probably because no reference NPs are available and the analytical methods for their characterization are more complex. Finally, industrially synthesized particles are non-polar and have a hydrophobic surface, but the aging of the plastic introduces functional groups that alter the polarity of the MPs and consequently also affect interactions with living cells, other chemicals, and organic matter. Therefore, future studies to evaluate the effects of MPs should consider different polymers, surface chemistry (for example, uncontaminated or degraded particles), different sizes and shapes, leaching of common additives, the presence of biological material such as microbial biofilms, and other exposure times to evaluate short- and long-term effects and different concentrations.
